# Fretting-corrosion Apparatus with Low Magnitude Micro-motion (≤5 μm): Development and Preliminary Outcome

**DOI:** 10.21203/rs.3.rs-3359897/v1

**Published:** 2023-10-05

**Authors:** Yani Sun, Kai-yuan Cheng, Hemalatha Kanniyappan, Remya Ampadi Ramachandran, Mozart Queiroz Neto, Michael McNallan, Robin Pourzal, Hannah Lundberg, Mathew T. Mathew

**Affiliations:** University of Illinois at Chicago; University of Illinois-School of Medicine at Rockford; University of Illinois-School of Medicine at Rockford; University of Illinois at Chicago; Rush University Medical Center; University of Illinois at Chicago; Rush University Medical Center; Rush University Medical Center; University of Illinois at Chicago

**Keywords:** Fretting apparatus, Fretting-corrosion, micromotions, hip implant

## Abstract

Fretting-corrosion is one of the failure processes in many applications, including biomedical implants. For example, the modern design of hip implants with multiple components offers better flexibility and inventory storage. However, it will trigger the fretting at the implant interfaces with a small displacement amplitude (< 5 µm) and usually in a partial slip region. Although many studies have been reported on the fretting, they have high displacement amplitude and are in the gross slip region. It is imperative to have an apparatus to overcome such limitations, specifically for hip implant applications. Therefore, this study describes the development of a fretting-corrosion apparatus with low micro-motion (≤ 5 µm) that can simultaneously monitor the corrosion process. Initial experiments with Ti6Al4V-Ti6Al4V in 0.9% saline, Ti6Al4V-Ti6Al4V in bovine calf serum (BCS), and ZrO_2_-Ti6Al4V in BCS were conducted to validate the system. As a result, the fretting regime of all groups remained partially slip region throughout the 3600 cycles, and the possible failure mechanisms are proposed in this manuscript.

## 1. INTRODUCTION

Fretting-corrosion is one of the failure processes in many applications, including biomedical implants[1]–[5]. For example, the modern Total hip replacement (THR) design with multiple components offers better flexibility and inventory storage. THR surgery is one of the most commonly performed procedures in the U.S. Currently, around 2.5 million people are living with hip replacements, and studies have shown that the number of individuals requiring THR is expected to increase to 4 million by 2030[6]–[8]. However, the lifespan of the current hip arthroplasty is typically between 10–20 years [9], implying that patients will likely have to undergo revision surgery and perhaps even re-revision surgery in the future. As reported before, revision surgeries have more significant associated risks, such as a more extended recovery period, surgery-related complications, fractures, and infections, at a diminished benefit for each revision[10][11]. Therefore, there is a great demand for well-functioning hip implants that maintain their integrity and functionality for as long as possible.

Studies show that there has been a surge in early hip implant failure due to wear-assisted corrosion (tribocorrosion)[12]–[15]. Fretting refers to the wear phenomena caused by oscillatory micromotion between two contact surfaces[4], [16], [17]. Previously, Brown et al. conducted *in-vitro* experiments on high corrosion-resistant surgical alloys, and it has been proved that the relative motion between these alloys can cause fretting-corrosion and release metal ions[18]–[20]. Also, earlier studies show that some subsequent damages, such as crevice corrosion, can be initiated by fretting[21]. As demonstrated in several reports, even though the design of currently used hip implants with modular head/neck junctions can allow better control of length, offset, and version, the modularity makes this location vulnerable to damages induced by micromotions at the interface[22]–[26]. As a result of the aggressive electrochemical environment of the body and the load simply being applied by patients’ physical weights, the oscillatory motion between head/neck junctions can be triggered as patients walk; fretting-corrosion will thus play a role and cause early hip implant failure.

In order to overcome this issue, it is necessary to simulate the *in vivo* fretting behavior first. To achieve that, the equipment will be required to simulate the *in vivo* environment and simultaneously evaluate the effects of mechanical wear and electrochemical reactions. Several efforts have been ongoing to investigate such equipment. For instance, Miyoshi et al. [27]–[29] evaluated the behavior of alloys and composites under fretting conditions by using a sliding/fretting wear apparatus, Geringer et al.[1], [2], [30] studied fretting-corrosion of orthopedic implant materials by a presented fretting device. Swaminathan et al. [31] proposed a fretting-corrosion test system to study CoCrMo and Ti6Al4V interfaces, and Royhman et al. [26] [32] designed a set-up and simulated fretting-corrosion in hip modular junctions. Nevertheless, some of these studies only focused on the mechanical aspects (micromotions) and ignored the response from the electrochemical side. In addition, the smallest displacement that can be currently achieved is 10–50 *μ*m[26], [32], [33], which might not be accurate enough to mimic the small micrometer motions occurring at the modular junction, which is less than 5*μ*m.

In this study, we report a device that is designed and manufactured to simulate a motion less than 5 *μm* between two contact surfaces while simultaneously providing feedback from electrochemical aspects with a final aim to evaluate the fretting-corrosion behavior of the hip implants. Preliminary experiments are conducted to examine the capability of the customized apparatus, which includes three groups: (i) metal-metal interface (Ti6Al4V-Ti6Al4V) in 0.9% saline as the initial trial run, (ii) metal-metal interface (Ti6Al4V-Ti6Al4V) in bovine calf serum (BCS), and (iii) ceramic-metal interface (ZrO_2_-Ti6Al4V) in BCS to investigate the performance of different contact couples in the simulated body fluids. After the fretting-corrosion testing, surface morphology was analyzed by scanning electron microscopy (SEM) and 3D profilometry.

## 2. MATERIALS AND METHODS

### 2.1 System description

#### (a) Basics of fretting and different fretting regions

**Figure 1:**
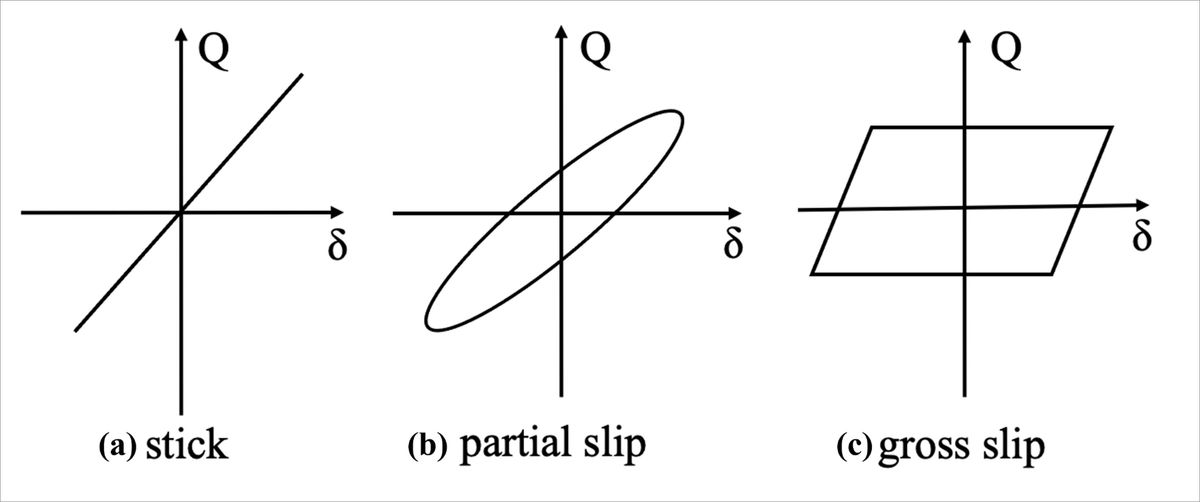
Illustration of three major fretting regimes: **(a)** stick, **(b)** partial and **(c)**gross slip. Q presents friction force, and δ presents displacements.

As briefly described before, fretting is a wear mode occurring under oscillating sliding conditions with a small amplitude (few microns) and high frequency. Researchers have been conducted on fretting wear and fatigue for more than 40 years[20], [34]–[38]. Typically, a ratio $\text{e}=\frac{\delta }{a}$ is used to define fretting, δ is the displacement amplitude and a is the contact size (width or radius of contact area), when e < 1, the motion is defined as fretting, otherwise it is considered as reciprocating sliding[36]. Depending on the amplitude of the displacements or on the normal load, fretting can be distinguished in three regimes: stick-slip, partial slip, and gross slip[34], [36]. In fretting tests, these three regimes can be identified by analyzing the tangential force (Q) and the displacement loop at each cycle. As displayed in Fig. 1, the corresponding Q-δ loops are closed, elliptic, or quasi-rectangular, respectively. If the shape of the fretting loop varies during the test, the regime is called a mixed fretting regime. Furthermore, Fouvry et al.[39] introduced a criterion for identifying the fretting regimes by energy ratios. The energy ratio is defined as the ratio of the dissipated energy in one cycle to the total energy lost in the whole test, and the fretting is considered a partial slip when the energy ratio is smaller than 0.2.

#### (b) Development of the fretting-corrosion system

This fretting-corrosion apparatus was developed for the specific application of hip modular junctions. The current features will enable researchers to study different material combinations, observe the electrochemical response, and monitor the changing friction over time. The details of the system will be described in this section.

##### (i) Mechanical aspects

**Figure 2:**
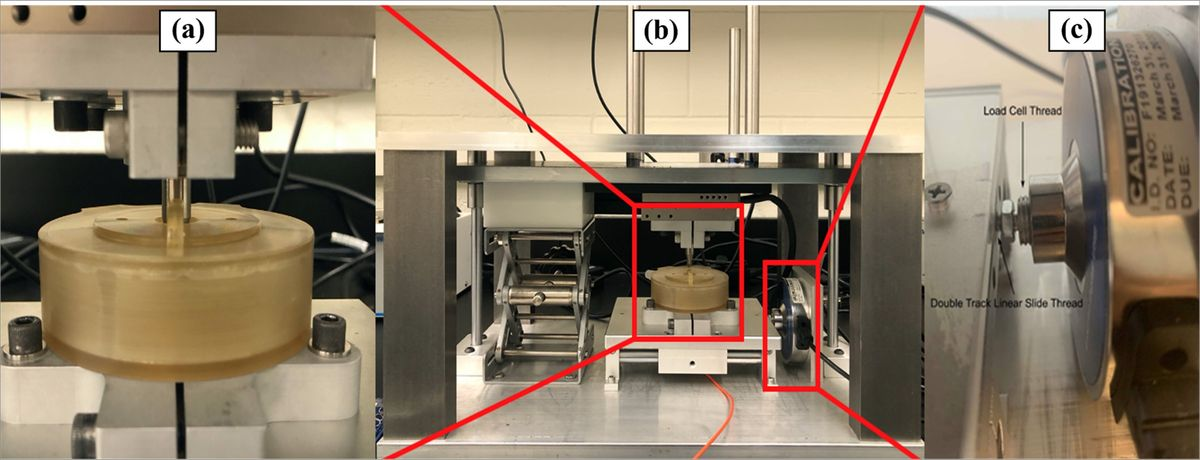
**(a)** the corrosion chamber, **(b)** Pictures of the actual fretting-corrosion apparatus and **(c)**the force sensor.

Figure 2 shows a general view of the actual fretting-corrosion apparatus. Frames made of 6061 aluminum were built to make the whole system more stable, which is considered an important factor in accurately detecting small movements. On top of the top plate, four pillars made of 6061 aluminum are used to support and stabilize the extra weights. 6061 aluminum was chosen since it has high corrosion resistance, high yield strength of 35,000 psi, and hardness of Brinell 95. The lab jack, linear bearings, and shafts allow the top plate to be lifted and lowered easily.

##### (ii) Actuator and load cell

**Figure 3:**
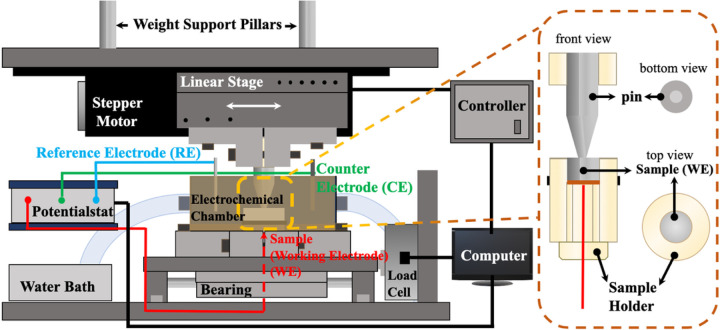
Schematic graph of the fretting-corrosion apparatus. A three-electrode set-up is used, and the system is connected to a potentiostat monitoring the electrochemical responses.

Figure 3 shows a schematic of our fretting equipment. The translation stage purchased from Thorlabs (NJ, USA) is combined with a stepper motor (DRV014) and a linear stage (LNR50SE) and attached to the top plate. According to the provided data sheet from the company, this translation stage can provide 50 mm travel and a minimum incremental movement of 50 nm, attributing to an integrated linear optical encoder and a compatible closed-loop stepper motor controller (BSC201). The linear optical encoder can give the necessary feedback to the electronic drive, and the closed-loop algorithm allows the system to move and maintain an encoded distance. The movement parameters, such as velocity, acceleration, and cycles, can be defined through the controller. The base plate has the double-track linear slide and load cell support mounted onto it. Bearings attached to the rods reduce friction between surfaces so that even small movements can be detected. A load cell purchased from LoadStar Sensors (CA, USA) is anchored to the double-track linear slide and utilized to detect and measure the tangential force we need to calculate the friction coefficient.

##### (iii) Pins, pin holder, and sample holder

The pin holder is fastened on the stage with a clamp, allowing the pin to move in a positive or negative x-axis direction with the linear stage. Three types of pins with different contact areas are available for users: (i) a conical pin with flat contact, (ii) a spherical pin with point contact, and (iii) a chisel pin with line contact. The sample holder made of polyether ether ketone (PEEK) can be fastened tightly on the slide by a clamp to avoid any possible interplay. In this preliminary study, we used a conical pin with 3mm dia, a flat surface in contact with the disc. The copper disk inserted in the holder contacts the sample that will be the working electrode.

##### (iv) Electrochemical chamber and connection

A designed double-wall electrochemical chamber can be assembled with a sample holder and mounted on the slide plate. The chamber can contain electrolytes such as bovine calf serum (BCS) and be connected to a water bath so that water with a certain temperature will go between the walls of the chamber and help it keep its temperature. Three inlets were made in the chamber lid for the pin, counter electrode, and reference electrode.

### 2.2 Preliminary study details

To test the capabilities of the customized fretting-corrosion system, three groups of experiments are designed with (i) Ti6Al4V on Ti6Al4V in 0.9% saline solution (TTS), (ii) Ti6Al4V on Ti6Al4V in BCS (TTB) and (iii) ZrO_2_ on Ti6Al4V in BCS (ZTB), as shown in Table 1. Each group of experiments was repeated three times (N = 3) to confirm reproducibility, and the results in this study are reported as the mean value with the standard deviation of three repeated experiments (N = 3). During the fretting phase, the pin was set to move 2 *μ*m reciprocally with a frequency of 1 Hz for 3600 cycles, and the electrochemical responses were monitored and recorded. After the fretting-corrosion testing, the element concentration in the solution was measured by inductively coupled plasma mass spectrometry (ICP-MS), and the sample surface was characterized by scanning electron microscopy (SEM) with energy dispersive spectroscopy (EDS) and 3D profilometry.

#### (a) Sample preparation

Ti6Al4V was selected as the material for the tested couple as it is one of the most commonly used biomedical materials. The Grade V Ti6Al4V was purchased from Supra Alloys, (CA, USA) and used as received. Before experiments, both Ti6Al4V pins (3 mm dia) and the Ti6Al4V disk (11 mm dia × 7 mm) were ground on the sandpapers (Buehler, USA) from grit 320 to 1200 and finally polished with the colloidal silica suspension (Buehler, USA) until the surface reached a mirror finish (Ra < 25 nm). ZrO_2_ was chosen as the other pin material to simulate the ceramic-metal interface. The ZrO_2_ pins were provided by the Argen Cooperation (CA, USA) and used as received. Also, the 0.9% saline solution was prepared by dissolving 9 g NaCl (Fisher Scientific, USA) in 1000 ml deionized (DI) water, and BCS (30 g/L) was utilized as the other testing electrolyte to mimic the body fluid.

#### (b) Evaluation of system compliance

**Figure 4:**
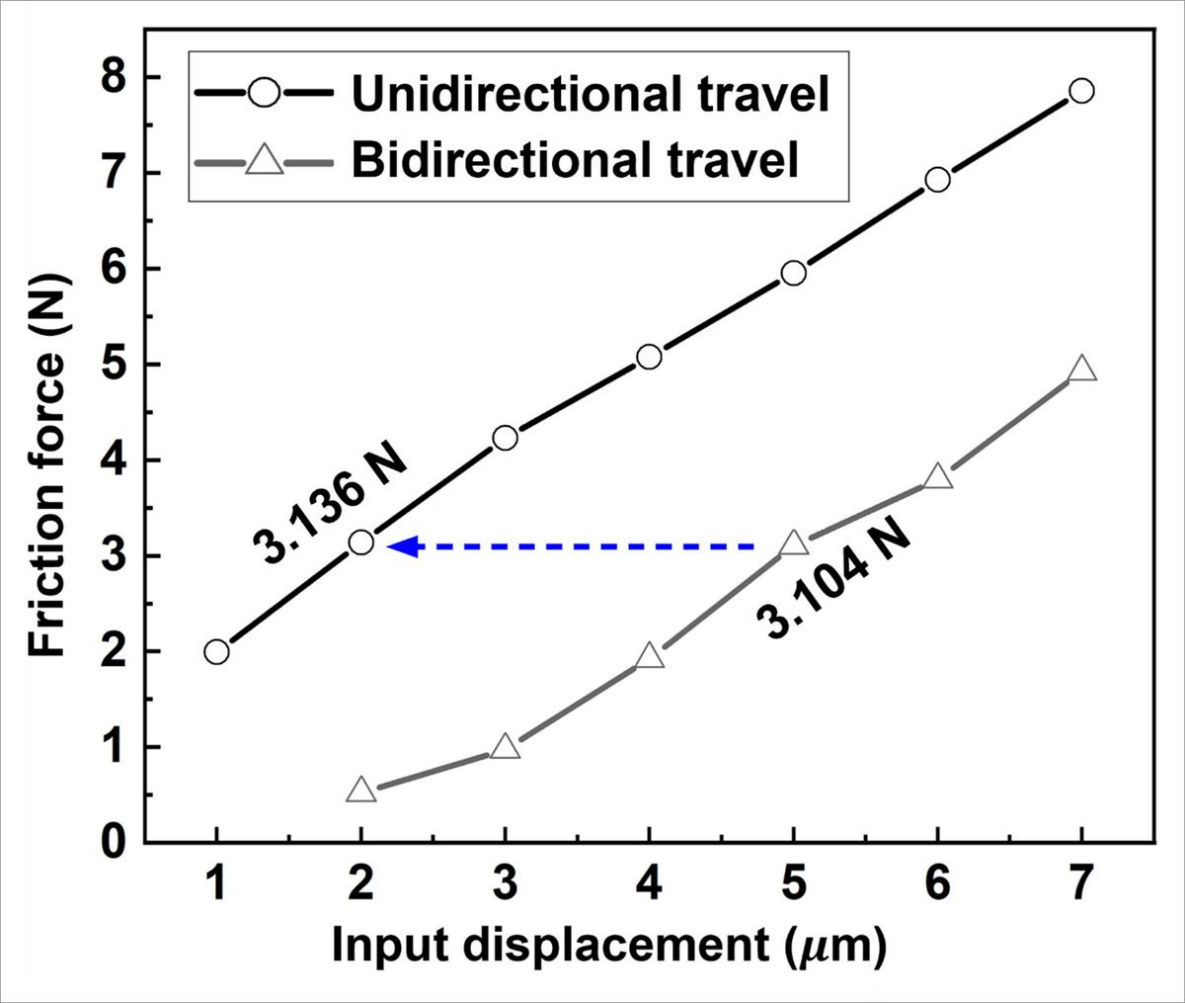
System compliance by the friction force correction. When the input is 5 μm, the generated friction force by birectional motion is 3.104 N, which is similar to the friction force created by unidirectional 2 μm motion (3.316 N), meaning that when the input is 5 μm, the actual movement at the interface is approximately 2 μm.

To evaluate the system compliance, an effort has been made to detect the actual displacements at the interface. However, no motion detectors or feasible techniques are discovered to directly measure such a micro-motion under 5 *μm*. Therefore, in this study, we utilized the friction forces at the interface to estimate the actual displacements. According to the specifications provided by Thorlabs Inc. (NJ, USA), the step motor (DRV014) can achieve a resolution of 50 nm with the bidirectional repeatability of less than 1 *μm*, so the bidirectional travel distance (oscillatory travel) is estimated based on the unidirectional travel (single travel). As shown in Fig. 4, the x-axis shows the input displacement, and the y-axis is the friction force generated at the interface by the motions. It can be seen that an input displacement of 5 *μm* under oscillatory travel creates a friction force of 3.104 N, which is close to the friction (3.136 N) generated by 2 *μm* under unidirectional travel. Therefore, under the oscillatory fretting motions, the pin moves on the disk approximately 2 *μm* back and forth when the input of 5 *μm* is given.

### 2.3 Fretting-corrosion test protocol

**Figure 5:**
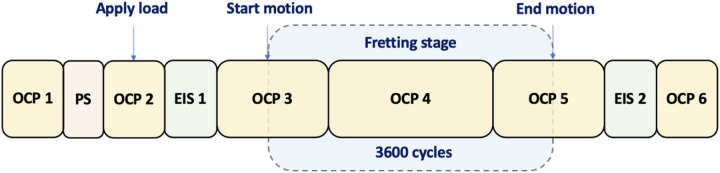
Illustration of the electrochemical protocol. Fretting stage includes 3600 cycles (3600 seconds) as labeled on the graph. EIS was conducted before and after the fretting stage. OCP-open-circuit potential, PS-potentiostatic, EIS-electrochemical impedance spectroscopy.

In the fretting-corrosion experiments, a normal load of 83 N is applied, and the pin is controlled to move with an amplitude of 2 *μ*m at 1 Hz for 3600 cycles. Besides, three electrodes are employed: the tested sample as the working electrode (WE), a graphite rod as the counter electrode (CE), and a Pt wire as the reference electrode (RE). After preparing the experiment materials, all electrodes are ultrasonically cleaned for 15 mins in isopropanol alcohol (IPA) and another 15 mins with DI water. Furthermore, the pin and the disk are assembled in the apparatus, and 20 ml solution is added to the corrosion chamber. As described in Section 2.2, a water bath is connected to the corrosion cell to maintain the temperature of the solution at 37±1°*C*. As shown in Fig. 5, a standard electrochemical protocol was utilized, which includes an open-circuit potential (OCP) stabilizing the testing environment, a potentiostatic (PS) cleaning the surface, and an OCP where the pin is loaded on the samples. Electrochemical impedance spectroscopy (EIS) examines the local impedance of the surface, another three OCPs record the potential evolution during the fretting phase, an EIS testing surface impedance after the fretting motions, and followed by a final OCP for the system stabilization.

### 2.4 Surface characterization

After the testing, samples are cleaned with the same procedures described in Section 2.3. In order to study the effects of the fretting-corrosion behavior on the sample, different techniques characterize the disk surface before and after the fretting-corrosion experiments. Bruker-Nano Contour GT-K Optical Profilometer is utilized to inspect the global surface changes in 3 dimensions and the surface roughness (Ra). A detailed surface morphology analysis was performed using a JEOL JSM-IT500HR SEM at 15kV, and Oxford Aztec EDS was used to acquire the elemental compositions.

## 3. RESULTS

### 3.1 Evolution of the fretting loops and the energy ratios

**Figure 6:**
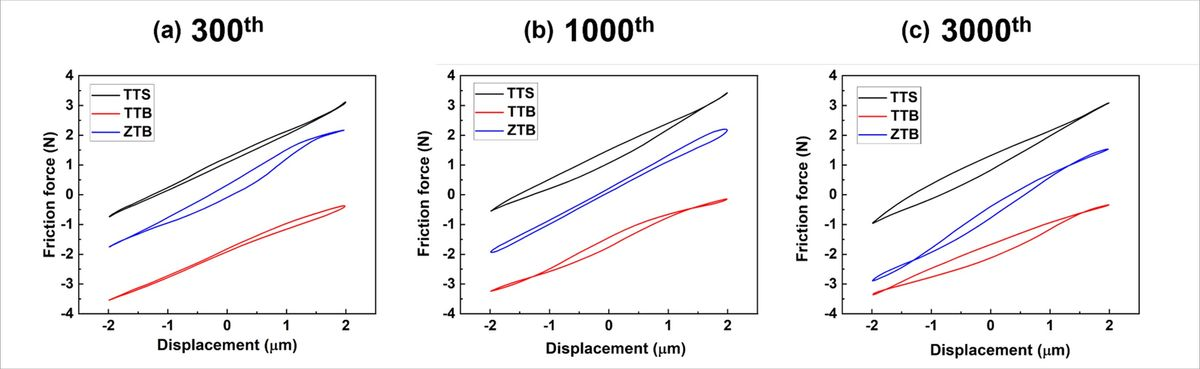
Fretting loops of TTS, TTB and ZTB at the **(a)** 300^th^, **(b)** 1000^th^, and **(c)** 3000^th^ cycles. All friction loops have a narrow elliptic shape, which is a typical indicator of partial slip.

The fretting loop is commonly presented as the tangential force versus the displacement. As shown in Fig. 6, the hysteresis loops of three groups are generated with the recorded friction force and displacement at the (a) 300th, (b) 1000^th,^ and (c) 3000th cycle, respectively. It can be observed that all loops have a narrow elliptic shape, which can be typically observed from a partial-slip regime as described in Section 2.1(a). Since the curve area of the loop presents dissipated energy, so the elastic deformation is dominant during the fretting movements for the partial-slip regime due to the narrow shape of the friction loops.

**Figure 7:**
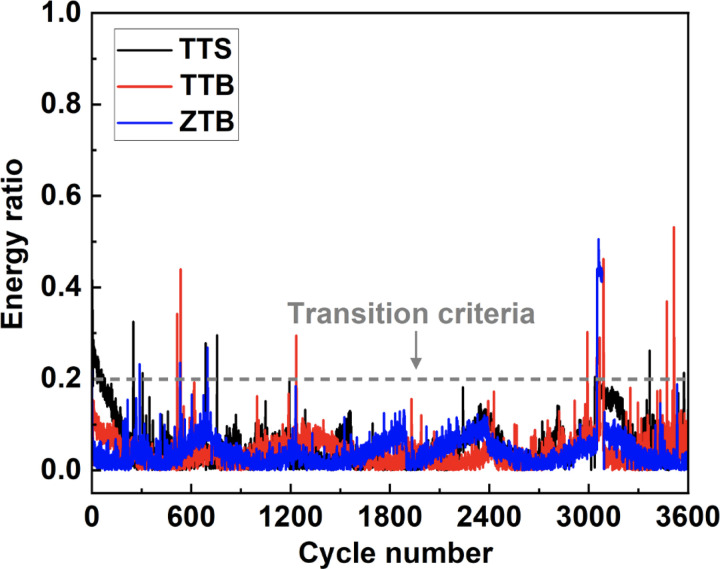
Evolution of energy ratios of TTS, TTB and ZTB. All energy ratios remain below 0.2 throughout the entire fretting stage, which is a direct indicator of partial slip.

Furthermore, according to Fouvry et al.[36], [39], the energy ratio is defined as the ratio of the dissipated energy to the total energy, which can be calculated as the ratio of the under-curve area to the whole area. Also, an energy ratio of 0.2 is considered a transition criterion for regime identifications, where the fretting is partial slip when the energy ratio is below 0.2 and gross slip when the ratio is larger than 0.2. In Fig. 7, the energy ratios of the three groups are calculated and presented. It can be observed that all energy ratios remained below 0.2 during the motion, which further confirmed a fretting regime of partial slip.

### 3.2 Evolution of open-circuit potential (OCP)

**Figure 8:**
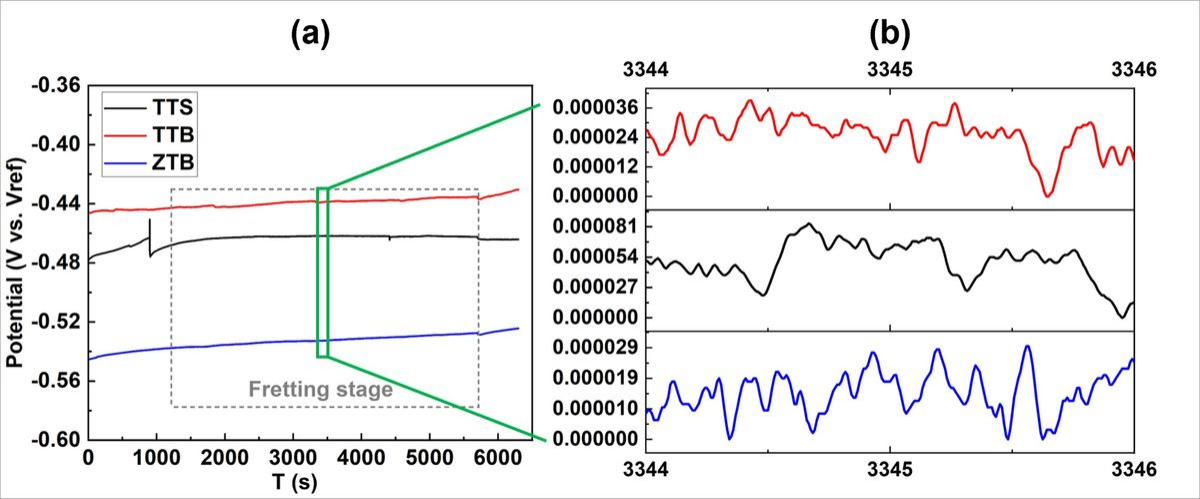
Evolution of energy ratios of TTS, TTB and ZTB. All energy ratios remain below 0.2 throughout the entire fretting stage, which is a direct indicator of partial slip.

Figure 8 displays the OCP curves of three groups, and the fretting stage is labeled with dashed lines. Generally, the OCP fluctuates cyclically when the pin moves on the titanium disk since the passive film on the titanium alloys would constantly be removed (de-passivation) and grow back (re-passivation) during motions. As can be seen in Fig. 8(a), TTS and TTB groups possess higher OCP than ZTB. Figure 8(b) presents the magnified section of the curve for four cycles; fluctuations caused by de-passivation and re-passivation can be seen, and the OCP variation of TTS is larger than that of TTB and ZTB, suggesting that TTB and ZTB groups are stable than TTS under the fretting-corrosion testing.

### 3.3 Electrochemical impedance spectroscopy (EIS)

**Figure 9:**
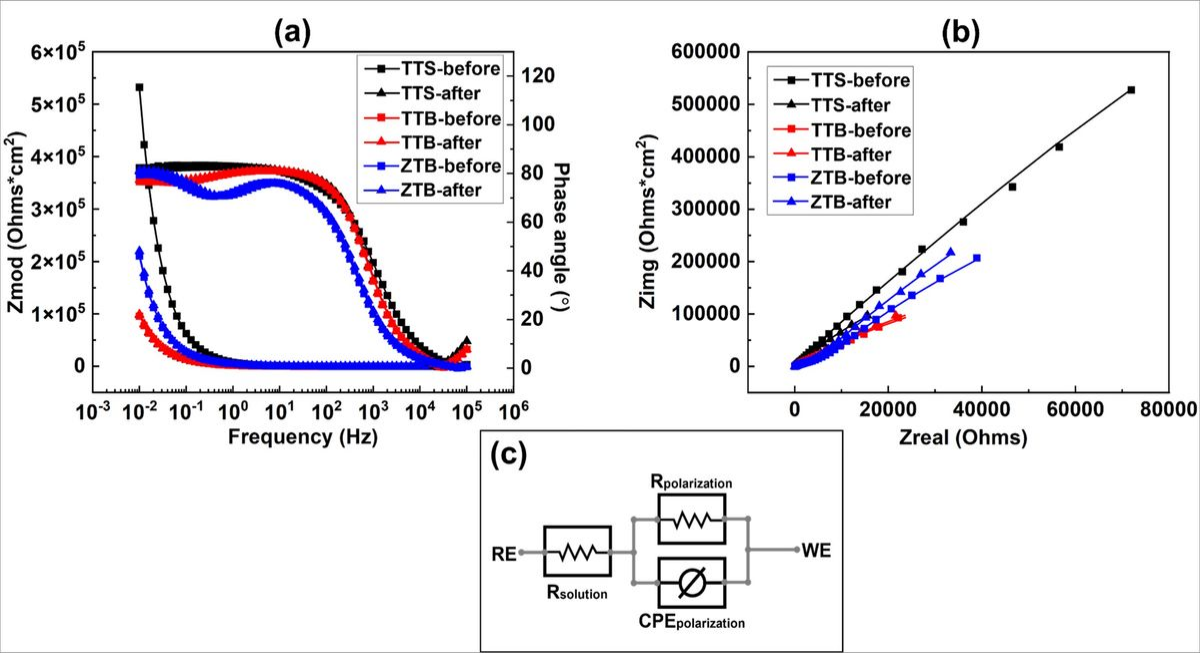
EIS results in **(a)** bode plots, **(b)** Nyquist plots where the scatter dots present original data and line plots present fitted results. **(c)**Equivalent circuit model – Randle modified with a constant phase element (CPE).

**Figure 10:**
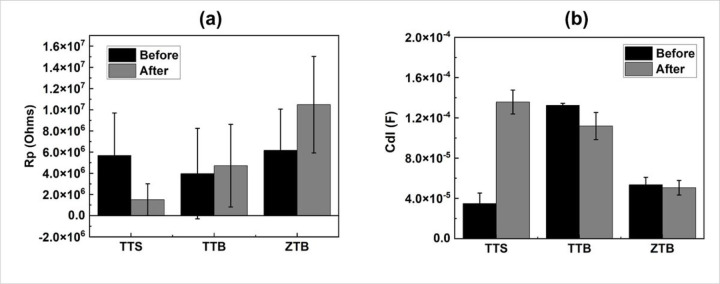
Bar diagrams of **(a)** the polarization resistance (Rp) and **(b)** the double-layer capacitance (Cdl). TTS group shows the lowest Rp and highest Cdl after the fretting-corrosion test.

EIS was included before and after the fretting stage to investigate the local resistance behavior. Figure 9 depicts the Bode plot **(a)** and the Nyquist plot **(b)** of the EIS results, where the scattering points present the raw data, and the lines denote the fitting results. A Randle circuit modified with a constant phase element (CPE) is used to fit the curves, as illustrated in Fig. 9(c). The fitted results of polarization resistance (Rp) and double-layer capacitance (Cdl) are organized in the bar diagrams as in Fig. 10. After fretting, Rp decreased and Cdl increased for TTS, whereas Rp increased and Cdl decreased for both TTB and ZTB groups. This finding is consistent with the OCP results and reveals that the existence of proteins may positively impact the fretting-corrosion behavior.

### 3.4 Surface characterization

**Figure 11:**
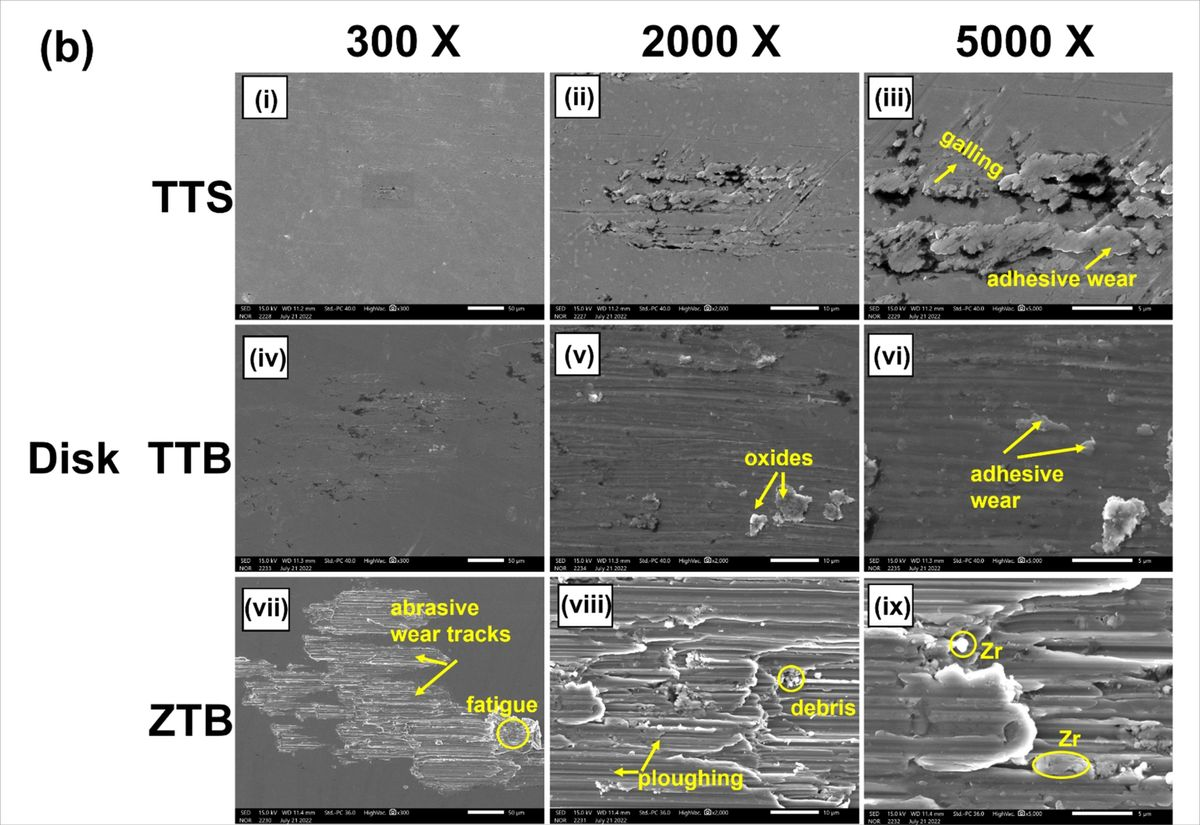
**(a)** SEM images of the pins from TTS, TTB, and ZTB. **(b)** SEM images of the Ti6Al4V disks from TTS, TTB, and ZTB groups. TTS and TTB generally show adhesive wear and ZTB mainly abrasive wear. More damage forms are marked on the graph.

**Figure 12:**
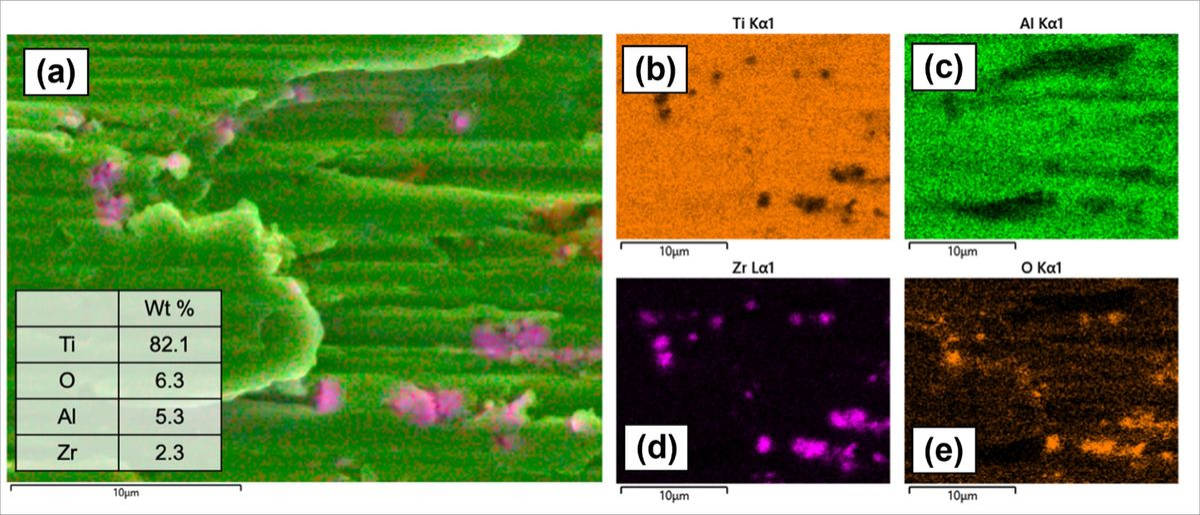
**(a)** Layered image of EDS mapping on the ZTB disk surface shows that ZrO_2_ is transferred to the Ti6Al4V disk after the fretting-corrosion test. **(b)** Ti **(c)** Al **(d)** Zr **(e)** O distribution.

**Figure 13:**
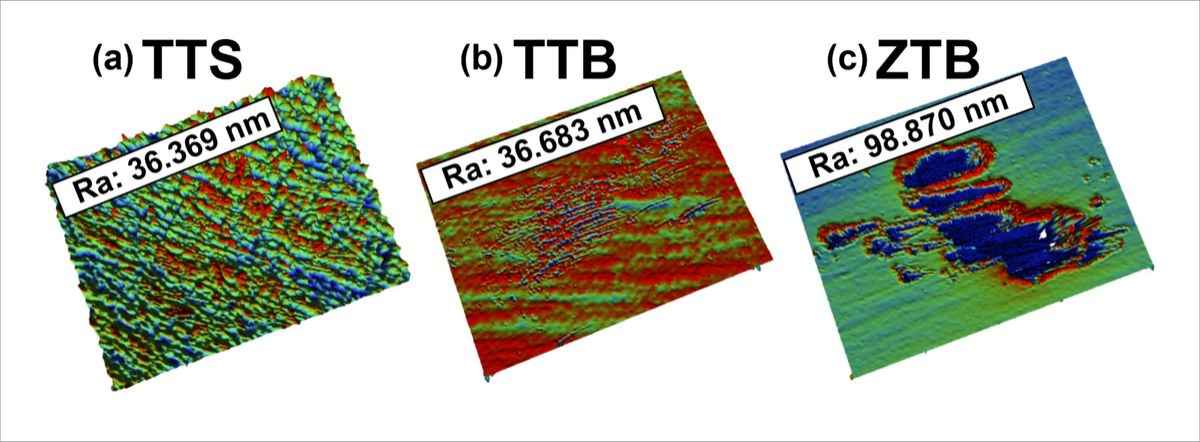
3D profilometry on **(a)** TTS, **(b)** TTB and **(c)** ZTB worn scars. Surface roughness values are labeled on the graph. TTS and TTB have similar Ra, however, Ra of ZTB is significantly higher (98.87 nm).

To analyze the surface after the fretting-corrosion experiment, SEM-EDS and 3D profilometry were employed. Figure 11(a) displays the SEM images of three pin tips, and Fig. 11(b) presents the surface of three disks. In Fig. 11a(i) and (iv), both TTS and TTB show a general fretting scar in parallel lines. Also, oxide accumulation was found on both TTS and TTB surfaces, as labeled in Fig. 11b(v). The disks of the ZTB group show the most severe damage among groups, and the scar mainly presents abrasive damage with plowing features. Surface fatigue can also be observed on this surface, as shown in Fig. 11b(vii). No obvious worn scar was observed on the zirconia tip as in Fig. 11a(vii–ix), which is expected since the hardness of zirconia is higher than Ti6Al4V. Interestingly, material transfer was observed on the ZTB disk, where zirconia was transferred to the Ti6Al4V disk based on the EDS results shown in Fig. 12. Similar results were discussed previously by Semetse et al.[30], which is believed that these detached ZrO_2_ particles would act as third-body particles and aggravate the wear at the interface [40]. Also, the 3D worn scars as well as surface roughness Ra of all groups after the fretting-corrosion testing, are displayed in Fig. 13. ZTB has a significantly higher Ra (98.87 nm), which might be because the hardness of ZrO_2_ is higher than Ti6Al4V. The wear volume was difficult to estimate from optical profilometry since some of the wear tracks are smaller than the polishing scratches, as can be seen in Fig. 13. Therefore, we employed ICP-MS to measure the metal ions released from the solution.

### 3.5 Metal-ion detection

**Figure 14:**
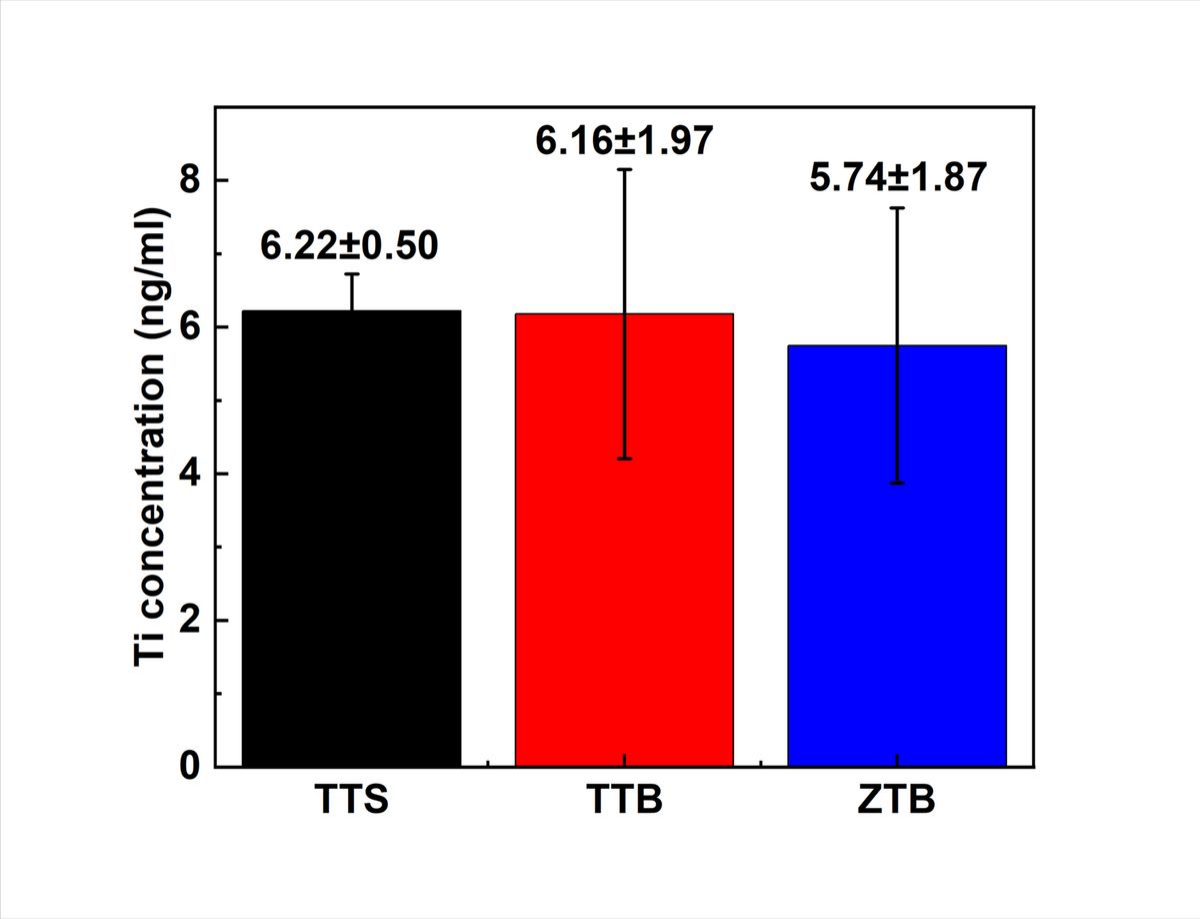
ICP-MS results show the released Ti concentration from TTS, TTB and ZTB after the fretting-corrosion testing. The Ti concentration detected in ZTB group is slightly lower than TTS and TTB.

Figure 14 displays the Ti ion concentration that existed in the solution after the fretting testing, and other types of ions are neglected as Ti is the main component (90%) of Ti6Al4V alloy. In general, groups with Ti6Al4V pins (TTS and TTB) released more Ti than ZTB group, which may be because the pins are made of zirconia in the ZTB group. Also, TTS possesses the highest Ti leaching concentration (6.22 ng/ml) among the three groups.

## 4. DISCUSSION

This study developed a new fretting-corrosion apparatus with a displacement amplitude of less than 2–5 µm, and preliminary results are reported.

### 4.1 Identification of partial slip fretting regions

The fretting regimes for the entire fretting process of all groups are identified as partial slip, which results from different aspects of support. Firstly, all fretting loops from the 300th, 1000th, and 3000th cycles shown in Fig. 6 have slim elliptic shapes, which is a typical indicator of partial slip as illustrated in Fig. 1. Secondly, according to the criteria reported by Fouvry et al.[36], [39], the regime is considered a partial slip when the energy ratio is below 0.2. As can be observed in Fig. 7, the energy ratios of all groups maintained below 0.2 for the whole duration. Combining all these findings, the fretting regimes of all groups can be identified as partial slip.

### 4.2 Fretting processes and Possible mechanisms

**Figure 15:**
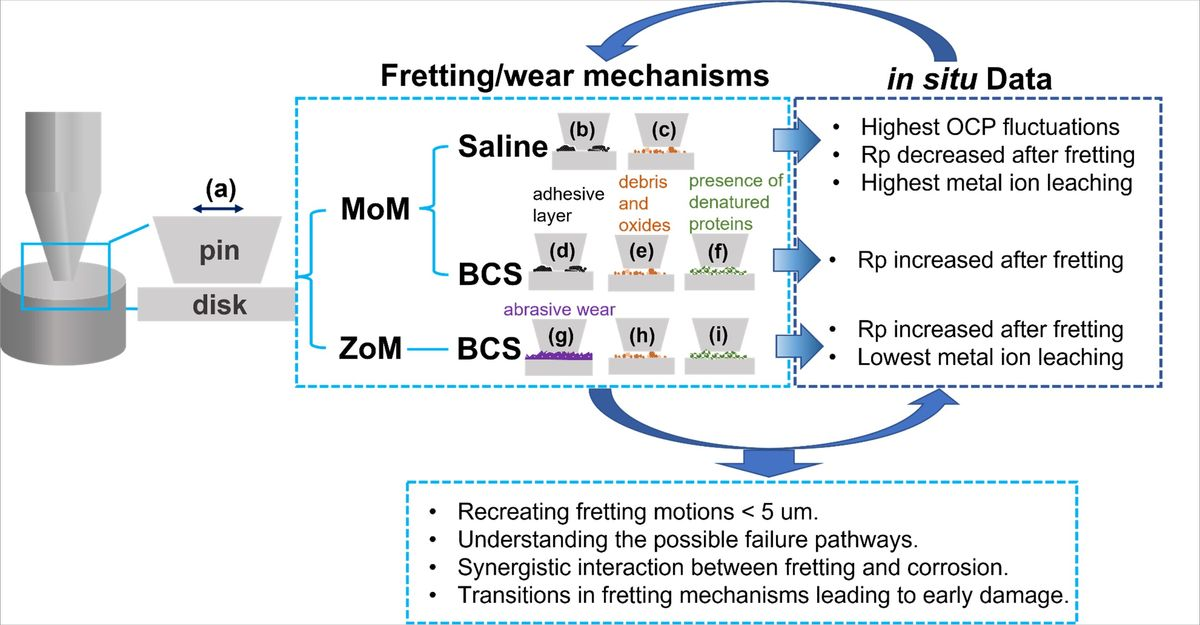
**(a)-(i)** Possible deformation paths under fretting-corrosion: **(b)&(d)**the adhesion wear from cold welding, **(c)(e)(h)**accumulation of wear debris and oxides **(f)&(i)** the presence of the denatured proteins.

Based on the results, possible deformation mechanisms are discussed in this section. As shown in SEM images in Fig. 11, the fretted surface of TTS and TTB show a feature of adhesive wear. It is known that Ti6Al4V has a propensity to gall, especially when the counter body is made of the same alloy[41]–[43]. Following this idea, one possible failure mechanism involved here might be parts of the pin (Ti6Al4V) and the disk (Ti6Al4V) cold-welded together at the interface, and the joint sections ruptured with the continuous fretting movements, leaving the adhesive layer on the surface, as illustrated in Fig. 15(b)&(d). A similar fretted Ti6Al4V-Ti6Al4V surface was also reported previously by Hager et al.[44].

Furthermore, several previous studies were found on the tribologically transformed structure induced by fretting[45]–[50]. According to these reports, the structure is formed by the accumulation of plastic deformation at the contact interface[44], and the produced layer has a similar Young’s modulus as the substrate alloy but a much higher hardness[50]. However, the layer may fracture abruptly under the loading and the movements, which can create third-body particles that scratch the surface and generate oxides. The third body debris and accumulated oxides may further damage the surface and cause material loss, as exhibited in Fig. 15 (c)&(e). In our SEM and EDS results, the worn debris of TTS and TTB surfaces were observed and identified as oxides, implying that the formation of tribologically transformed structures might also be applied here to explain the failure mode. Finally, these two failure mechanisms might co-exist in the process and result in the final worn surface.

On the ZTB group, parallel scratches were mainly observed on the worn surface, which indicates the abrasion wear with typical ploughing features as in Fig. 15 (g), and it is evident that ZrO_2_ was transferred to the alloy surface under the fretting motions according to the EDS results. Previously, Semetse et al.[30] investigated the fretting-corrosion performance of Ti6Al4V enforced with ZrO_2_ and also discovered the ZrO_2_ particles on the counter body. It is believed that the detached hard debris would act as a third body and further scratch the counter surface, as in Fig. 15 (h), leading to more severe wear at the interface[40]. Also, Feyzi et al.[51] The tribocorrosion properties of ZrO_2_ ball rubbing against the Ti6Al4V disk were studied, and similar worn scars and material transfers were reported. Moreover, surface fatigue can also be seen on the Ti6Al4V disk (in the ZTB group), which might be formed due to the cyclic oscillations.

### 4.3 Comparison with other reported studies on fretting-corrosion

Our preliminary results are compared with previously reported studies in this section to validate our fretting-corrosion system. Previously, Royhman et al.[26] developed an apparatus to investigate the fretting-corrosion in hip implant modular junctions and conducted the initial experiments with Ti6Al4V-Ti6Al4V with displacement amplitudes of 50 µm for 1400 cycles. According to their results, the energy ratio maintained below 0.2 for 1400 cycles when pH was 7.6, but it increased with the cycle numbers and nearly reached 0.2 at the end of the testing. In our case, the energy ratio also remained less than 0.2 for 3600 cycles and did not show an obvious increasing tendency. Also, the dissipated energy for the first 1400 cycles of our work was calculated to be 0.0319 J (TTB), which is approximately 0.53% of the reported data. These differences were within the expectation since the amplitude used in this study is only 25-fold smaller than 50 µm. Furthermore, compared to the results reported by Klekotka et al. [52], our OCP evolution showed fewer fluctuations, and the worn surface had less damage despite the fact that a similar adhesive wear mode was observed, which might also be because the researchers investigated the fretting performance under a much larger movement amplitude (100 µm). Even though these previous studies were conducted under different conditions, comparisons made in this section are to confirm that the results derived from our customized set-up are reasonable and valid. In the future, it is still necessary to design a series of experiments to evaluate the novel micromotion fretting-corrosion system fully.

### 4.4 Limitations of the apparatus and future directions

Finally, this work has several possible limitations and related future studies are proposed in this section. The first aspect is the accurate measure of the real contact area, which is generally a difficult task when a flat-on-flat tribological contact is employed. Although a contact pressure is calculated to be 11.74 MPa with a force of 83 N and a geometrical pin tip area of 7.07 mm^2^, the real contact pressure might be much higher since the contact area is smaller than the geometrical area and plastic damages occurred based on the SEM images. With this contact condition, this fretting-corrosion apparatus might not be capable of simulating the actual case at the modular junction of hip implants; however, we plan to use a pin-on-ball (point contact) configuration or pin-on-flat (pin has chisel edge) to achieve a higher measurable contact pressure in the following study. Secondly, further study is required for system compliance of the apparatus. The current system has only minimum mechanical interfaces; however, the tolerance from different components may affect the actual displacement at the sample interface. Although the displacement at the interface was corrected and verified for this study, the process to comply with the system needs to be done for different testing conditions, such as different testing couples, different solutions, and additional loadings. In future studies, more efforts will be made to improve this drawback. For instance, the pin holder is currently made of PEEK to insulate the equipment from electrical conductions; it might be better to fabricate the holders with other materials with a higher Young’s modulus. The sample couples were used in this study only for the preliminary investigations; more relevant materials couples will be considered in the next stage of validation. Furthermore, the duration of experiments in this study was chosen as 3600 cycles, and we plan to conduct long-term experiments to validate the apparatus. Lastly, as a purpose of validating the system, we utilized ZrO_2_ as the ceramic counter-body; however, it is not widely used to manufacture the femoral head of hip implants. In the following study, we will study the materials that are more relevant to hip prosthesis, for instance, Al_2_O_3_.

## 5. CONCLUSIONS

In this study, a customized fretting-corrosion apparatus was designed and manufactured to investigate the fretting behavior (≤ 5 um) with simultaneous electrochemical feedback, and a series of preliminary fretting experiments was carried out with Ti6Al4V on Ti6Al4V in 0.9% saline solution for 3600 cycles to verify the system. Therefore, the main conclusions from this study are:

- Typical partial slip fretting loops were observed, and the energy ratios remained below 0.2 throughout the entire fretting stage.
- On the electrochemical side, the fluctuations due to the de-passivation and re-passivation were shown on the OCP curve, and TTS shows larger OCP variations than TTB and ZTB, which might be because of the absence of proteins in the saline solution. Also, the local resistance decreased for TTS but increased for TTB and ZTB after the fretting phase based on the EIS results, which suggests that the existence of protein might have a positive impact on the fretting-corrosion performance.
- The deformation occurred potentially due to two paths: the adhesion wear from cold welding and the material loss from the tribologically transformed structure.
- The material couples used in this preliminary study are Ti6Al4V on Ti6Al4V and ZrO_2_ on Ti6Al4V; however, more relevant material couples for hip implant application (Ti6Al4V on CoCrMo, CoCrMo on CoCrMo, Al_2_O_3_ on Ti6Al4V, and Al_2_O_3_ on CoCrMo) should be selected for the future work.
- This is an ongoing study focusing on fretting-corrosion behavior with micromotions below 5 µm and simulating the actual contact pressure at the hip taper junction.

## Figures and Tables

**Table 1:** Details of the sample groups

	Pin	Disk	Solution
**TTS**	Ti6Al4V	Ti6Al4V	0.9% saline
**TTB**	Ti6Al4V	Ti6Al4V	BCS
**ZTB**	ZrO_2_	Ti6Al4V	BCS
